# Exploring the Association between Negative Emotions and COVID-19 Vaccine Acceptance: A Cross-Sectional Analysis of Unvaccinated Adults in Sweden

**DOI:** 10.3390/vaccines10101695

**Published:** 2022-10-11

**Authors:** Ying Wei, Nigel Walsh Harriman, Rachael Piltch-Loeb, Marcia A. Testa, Elena Savoia

**Affiliations:** 1 Emergency Preparedness Research, Evaluation & Practice Program, Harvard T.H. Chan School of Public Health, 677 Huntington Avenue, Boston, MA 02115, USA; 2 Department of Biostatistics, Harvard T.H. Chan School of Public Health, 677 Huntington Avenue, Boston, MA 02115, USA

**Keywords:** COVID-19, vaccination, emotions, psychology, financial stress, government

## Abstract

The coronavirus disease 2019 (COVID-19) pandemic has had a significant impact on individuals’ mental health. This study aimed to investigate how negative emotions toward the COVID-19 pandemic, including feeling anxious, depressed, upset, and stressed, were associated with COVID-19 vaccine acceptance in Sweden. The study is a cross-sectional online survey conducted between 21–28 May 2021, using three nested hierarchical logistic regression models to assess the association. The study included 965 unvaccinated individuals, 51.2% (n = 494) of whom reported their intention to get vaccinated. We observed graded positive associations between reported negative emotions and vaccine acceptance. Individuals who experienced economic stress had lower odds of vaccine acceptance while having a positive opinion of the government’s response to COVID-19 was associated with higher odds of being vaccine-acceptant. In conclusion, unvaccinated individuals experiencing negative emotions about the pandemic were more willing to get the vaccine. On the contrary, those with a negative opinion about the government’s response, and those that had experienced economic stress were less likely to accept the immunization.

## 1. Introduction

The coronavirus disease 2019 (COVID-19) pandemic has had an unprecedented impact on health, economic, social, and political systems around the world. As reported by the most recent WHO European Health Report, the social restrictions imposed during the COVID-19 pandemic, such as schools and business closures and enforced social distancing measures, have had a profound effect on populations’ mental health, with a surge of people suffering from loneliness, anxiety, and depression [1]. Existing scientific literature has highlighted the negative psychological effects of pandemic stressors such as long quarantine periods, fear of infection, depression, boredom, difficulty in finding daily goods and medications, insufficient information, and financial loss [2]. Furthermore, individuals have also indicated general concerns about the duration of the pandemic, employment and economy, and overall uncertainty about their future [2,3]. In addition, information overload and exposure to misinformation have negatively impacted people’s attitudes and emotions [4]. People also developed some unhealthy behaviors during the COVID-19 pandemic, such as a decrease in physical activity and walking, an increase in weight, smoking, and worsening sleep and dietary habits [5]. The rate of vaccination is critical to controlling the COVID-19 pandemic as herd immunity is effective in slowing the spread of an epidemic. A proactive attitude toward vaccination and fair recognition of disease risk can also influence the dynamics of COVID-19 spread [6]. As of August 2022, Sweden has reported over 2.5 million confirmed COVID-19 cases and over 19,000 related deaths, but 24% of the population were still not fully vaccinated [7]. Studies have found that sociodemographic characteristics (including age, sex, employment status, educational level, and economic stress), racial discrimination, and misinformation are associated with COVID-19 vaccine hesitancy [8,9,10,11]. Knowledge about the disease and vaccine attitudes also affect willingness to pay for the vaccine [12]. Extant research has indicated that increased knowledge of disease (like treatment and vaccine) was significantly associated with higher risk perception and a higher probability of practicing preventive behavior [13]. However, few extant studies have investigated the relationship between psychological states and COVID-19 vaccine hesitancy [14,15,16].

Recent studies have indicated that over a quarter of the Swedish population experienced symptoms of depression, anxiety, and insomnia during the crisis, approximately twice the observed prevalence before the start of the pandemic [17,18]. The prevailing negative sentiments related to COVID-19 vaccination include criticism of vaccine mandates and regulations, risk of side effects, and skepticism about its effectiveness [19]. In the process of vaccine dissemination and education, an important aspect of communication is to consider the role of psychological states. Studies have shown that emotional engagement is a critical component of behavior change, but the relationship is not straightforward. In some anti-vaccination campaigns, heightened emotions (such as anger) were used to promote misinformation and conspiracy theories, resulting in vaccine hesitancy [14]. Existing research has demonstrated that the experience of stress hampers compliance with prescribed medical treatment [20]. Researchers have also begun to study this stress and compliance association concerning compliance with recommended measures to reduce the spread of infectious diseases [21]. As for COVID-19, however, there is some evidence that individuals who are worried about becoming infected tend to be diligent and compliant with containment measures [15]. This finding is consistent with fear appeals theory under the framework that fear appeal communication attempts to influence or persuade changes in behavior through the threat of impending danger or harm [22,23]. Another study indicated that greater perceived moral reproach–the feeling of being judged as immoral for being unvaccinated-was independently associated with COVID-19 vaccine hesitancy [16]. Given the scant attention given to the topic by the scientific community, and its potential to impact health behavior change, the association between psychological states and COVID-19 vaccine acceptance should be further explored.

Unlike most countries, which enforced widespread sector closures and strict social distancing measures through legal mandates with punishments, the Swedish government took a non-legally mandated approach based on community education, personal choice, and social responsibility, rather than on government enforcement [24,25]. Given that psychological states are associated with shaping behavior, the current study aims to (1) investigate how COVID-19 vaccine acceptance in Sweden is related to negative emotions, including feeling anxious, depressed, upset, and stressed about the COVID-19 pandemic situation, and (2) explore the association between COVID-19 vaccine acceptance, economic stress, and opinions about the government’s response to prevent the spread of disease.

## 2. Materials and Methods

### 2.1. Study Design and Data Collection

Our analysis uses a cross-sectional study design with data derived from an online survey collected via mobile devices on the Pollfish survey platform. Pollfish pays mobile application developers to display and promote the surveys to the users within their applications. To incentivize participation, small monetary reimbursements are provided to randomly selected users who complete the surveys. Pollfish uses random device engagement (RDE) to reach users of mobile applications who are identified only by a unique device ID. An initial survey instrument draft was developed and given to 20 individuals who spoke English and Swedish for cognitive testing and to assess the face validity of the survey items. Subsequently, the survey was revised according to their feedback to ensure that it could be intelligibly translated into Swedish before implementation. Individuals were eligible to participate in this study if they were over 18 years of age, living in Sweden, and had not yet been vaccinated. A random sample of users who fit the study’s eligibility criteria was initially selected and data were collected between 21–28 May 2021 (n = 1000). The sample had equally distributed quotas of respondents by sex and age group (18–24 years, 25–34 years, 35–44 years, 45–54 years, and 55+ years). As a data quality assurance method, it was estimated that it would take at least 3 min to complete the survey. Therefore, we removed from the analysis individuals who completed the questionnaire in less than 3 min (n = 35). This resulted in a final sample of 965 respondents, with an average completion time of 10 min 20 s. The study was approved by the Harvard T.H. Chan School of Public Health Institutional Review Board (protocol number IRB20-2032). The questionnaire was voluntary and consented to by the participants.

### 2.2. Dependent Variable (Vaccine Acceptance)

Participants were asked about their willingness to get vaccinated if they were offered the COVID-19 vaccine at no cost within two months. Response options were: (1) very likely, (2) somewhat likely, (3) I am not sure, (4) somewhat unlikely, (5) very unlikely, and (6) I would not take it at the moment but would consider it later on. The responses were dichotomized into two categories (1 = “Vaccine-Acceptant”, if the answer “very likely” was chosen, and 0 = “Vaccine-Hesitant” if any other response was chosen).

### 2.3. Independent Variables

The primary independent variable of interest was the degree of negative emotions toward the COVID-19 pandemic situation. Respondents were asked to rate how much they agreed with the following 4 statements: (1) I feel anxious when I see the number of COVID-19 cases climbing; (2) I feel depressed about the uncertainty of how this pandemic will evolve; (3) I get upset when I hear contradictory information about COVID-19; (4) I feel stressed when I am unable to plan my life due to COVID-19. Responses were given using a Likert scale ranging from 1 = not concerned to 3 = very concerned. 

The factor structure of the four items was assessed using principal component factor analysis. A scale with a score ranging from 4 to 12 was formed, with higher values indicating stronger negative emotions toward the situation. The suitability of the data for factor analysis was assessed using the Kaiser-Meyer-Olkin (KMO) measure of sampling adequacy and Bartlett’s test of sphericity. Cronbach’s alpha reliability coefficient was used to measure the internal consistency of the scale. The scores were categorized into three groups according to tertiles: low (≤33rd percentile, 4–7), medium (>33rd percentile–≤67th percentile, 8–9), and high (>67th percentile, 10–12) degree of negative emotions.

Table 1 presents data on participants’ sex, age, employment status, educational level, economic stress, number of comorbidities, previous COVID-19 diagnosis, past refusal of other types of vaccine, opinion about the government’s response to the pandemic, and degree of negative emotions related to their experience of the COVID-19 pandemic. More specifically, economic stress was measured by the dichotomous response (Yes or No) to the question: “*Have there been occasions in which you were worried about not having enough money or resources to be able to have enough food to eat in the past 12 months*”. Comorbidities included: cancer, severe allergies, seizures, being in an immunocompromised state, obesity, diabetes, cardiovascular disease, hypertension, pulmonary disease, and rheumatological conditions. To capture their opinion of the government’s response to COVID-19, participants were asked if they thought the measures taken by their government to respond to the pandemic had been appropriate. 

### 2.4. Statistical Analysis

Descriptive statistics were performed to describe the sample characteristics. Chi-squared tests were used to examine the univariate relationships between the categorical covariates and COVID-19 vaccine acceptance. We then performed principal component factor analysis to explore the factor structure of the negative emotions scale. Subsequently, we assessed the association between negative emotions and vaccine acceptance in three nested hierarchical logistic regression models using the following method: model 1 included participant demographic variables only (sex and age); model 2 included all the parameters in model 1, plus proxies for socioeconomic status (education, employment status, and economic stress); and model 3 included the parameters from model 2 plus comorbidities, COVID-19 diagnosis, past vaccination refusal for other types of vaccines, and opinions about the government’s response to the pandemic. Predictive analysis for each model was measured by calculating the area under the receiver operating characteristic curve (AUC) and Akaike information criterion (AIC). The Hosmer-Lemeshow test was used to evaluate the goodness of fit of each model. The type 1 error (α) for rejecting the null hypothesis was set at 0.05. Analyses were carried out using Stata Statistical Software: version 17, StataCorp LLC, College Station, TX, USA. 

## 3. Results

### 3.1. Sample Characteristics and Descriptive Statistics

Baseline characteristics were summarized in Table 1. Our data included 965 respondents, 51.2% (n = 494) of whom reported vaccine acceptance. The distributions of sex, employment status, and educational level were similar between the acceptant group and the hesitant group (*p* > 0.05). Vaccine-acceptant participants less frequently reported economic stress and they felt that the government’s response to the COVID-19 pandemic was appropriate (*p* < 0.05). Individuals of older age and with one or more comorbidities were more likely to be vaccine-acceptant (*p* < 0.05). Conversely, people with previous COVID-19 diagnoses and past vaccine refusal were more likely to be vaccine-hesitant (*p* < 0.05). 

### 3.2. Factor Analysis and Descriptive Statistics of Negative Emotions toward the COVID-19 Pandemic

The overall KMO measure of sampling adequacy (0.765) and Bartlett’s test for sphericity (*p* < 0.001) indicated that the items measuring negative emotions were suitable for factor analysis. Principal components factor analysis of the four items resulted in one factor with an eigenvalue greater than 1 (57.14% variance explained), and all factor loadings were greater than 0.73 (Supplementary Table S1). Responses to the four items were summed to generate a scale with a mean of 8.2 (standard deviation 2.2) and a median of 8 (range 4–12). The Cronbach alpha for the scale items was 0.75. Chi-squared results indicated as the negative emotion degree increased, people tended to be more accepting of the COVID-19 vaccine (low degree vs medium degree vs high degree, 45.4% vs. 51.7% vs. 57.6%, *p* = 0.009) (Table 1).

### 3.3. Multivariate Logistics Regression Analysis

**Figure 1:**
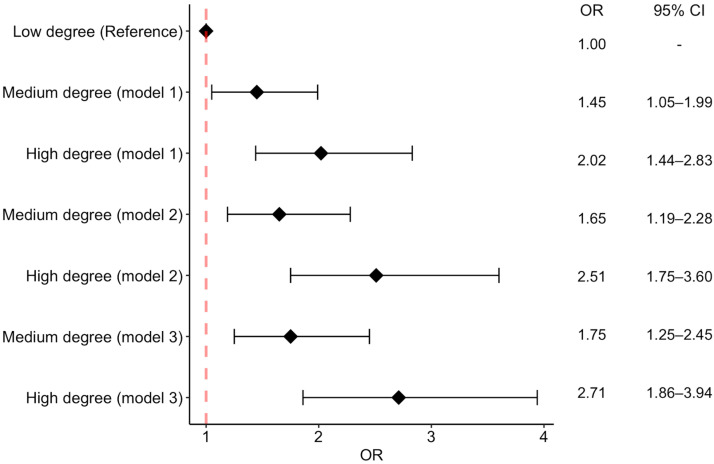
ORs between negative emotion degrees and COVID-19 vaccine acceptance. OR: odds ratio, CI: confidence interval. Model 1: Adjusting for demographic factors (including sex and age). Model 2: Adjusting for demographic and socioeconomic status factors (including education, employment status, and economic stress). Model 3: Adjusting for demographic and socioeconomic status factors, and other risk factors (including comorbidities, COVID-19 diagnosis, vaccination refusal in the past, and opinions about the government’s COVID-19 response).

We analyzed the association between negative emotions and the outcome separately in three models (Table 2 and Figure 1).

*Model 1. Adjusting for demographic factors*

In model 1, after adjusting for demographic factors (sex and age), participants with a medium degree of negative emotions had 45% increased odds of being vaccine-acceptant (OR = 1.45, 95% CI: 1.05–1.99), and participants with a high degree of negative emotions had 102% increased odds of being vaccine-acceptant (OR = 2.02, 95% CI: 1.44–2.83), compared to those with a low degree of negative emotions.

*Model 2. Adjusting for demographic and socioeconomic factors*

Model 2 added socioeconomic status factors (education, employment status, and economic stress) to the parameters included in model 1. Compared to those with a low degree of negative emotions, participants with a medium degree of negative emotions had 65% increased odds of being vaccine-acceptant (OR = 1.65, 95% CI: 1.19–2.28), and participants with a high degree of negative emotions had 151% increased odds of being vaccine-acceptant (OR = 2.51, 95% CI: 1.75–3.60). Participants who experienced economic stress had 54% lower odds of being vaccine-acceptant, compared to those who had not (OR = 0.46, 95% CI: 0.34–0.62).

*Model 3. Adjusting for demographic, socioeconomic factors, and other risk factors*

In model 3, we added the following parameters to model 2: comorbidities, COVID-19 diagnosis, vaccination refusal in the past, and opinions about the government’s COVID-19 response. Compared to participants with a low degree of negative emotions, participants with a medium degree of negative emotions had 75% increased odds of being vaccine-acceptant (OR = 1.75, 95% CI: 1.25–2.45), and those with a high degree of negative emotions had 171% increased odds of being vaccine-acceptant (OR = 2.71, 95% CI: 1.86–3.94). Participants who experienced economic stress had 57% lower odds of being vaccine-acceptant, compared to those who had not (OR = 0.43, 95% CI: 0.31–0.59). Participants who thought the government’s COVID-19 response was “just right” had 148% increased odds of being vaccine-acceptant (OR = 2.48, 95% CI: 1.80–3.40), compared to those who believed that the government’s response was either excessive, not useful, counter-productive, or were unsure about its effectiveness. 

Hosmer-Lemeshow Goodness of Fit test results confirmed that the final model was a good fit for the data (*p* = 0.97). This model had an AUC of 0.723 (95% CI: 0.691–0.755). Furthermore, the addition of negative emotions to the established models significantly increased AUC (*p* < 0.05), and reduced AIC in model 1, model 2, and model 3, separately (Table 3).

To assess whether the association between negative emotion and vaccine acceptance varied by age, we reproduced the fully adjusted model stratified by age group (adjusted by sex, employment status, educational level, economic stress, comorbidities, previous COVID-19 diagnosis, past refusal of other types of vaccine and opinion about the government’s response to the pandemic) (Supplementary Table S2). The results also indicated a positive dose-dependent association between negative emotions and vaccine acceptance, with a *p*-value (likelihood-ratio test) of 0.66 for the interactive effect of age, suggesting that the association between negative emotion and vaccine acceptance was not significantly different across these age groups.

## 4. Discussion

Few studies have focused on COVID-19 vaccine acceptance in Sweden [11,26,27,28]. To our knowledge, this study is the first to explore the relationship between negative emotions toward the COVID-19 pandemic situation and vaccine acceptance in a sample of unvaccinated individuals living in this country. This study also investigates the association between vaccine acceptance, economic stress, and opinions about the government’s response, while controlling for sociodemographic and health determinants. As expected, our results indicate that people who are more likely to experience severe consequences from the infection, such as those ≥45 years and those with comorbidities are also more willing to get vaccinated [29,30]. In addition, similar to other studies, our results show that having a previous COVID-19 diagnosis and past refusal of other vaccines are associated with lower COVID-19 vaccine acceptance [10,31]. 

Regarding our major variables of interest, our findings indicate a positive dose-dependent association between negative emotions about the pandemic (expressed as feeling anxious, depressed, upset, and stressed) and COVID-19 vaccine acceptance. There are conflicting conclusions about the relationship between emotions and compliance with recommendations. Our result provided data support for the effect of psychological factors on non-adherence to vaccination and hygiene programs. Attention to emotion could complement other major factors that influence vaccine education. However, our result might seem contrary to what other researchers have theorized-that soliciting negative emotions in communities with pervasive heightened emotions is likely to have the opposite effect, making people afraid and less willing to get vaccinated [14]. On the contrary, another study indicated that threat intervention and prosocial intervention both increased willingness to self-isolate during the COVID-19 pandemic, and the efficacy of the prosocial intervention depended more on the strength of the emotional response compared to the threat intervention [32]. The emotional experiences in that study were measured on an unpleasant-pleasant dimension (i.e., valence) and a low-high activation dimension (i.e., arousal), which was different from our study (feeling anxious, depressed, upset, and/ or stressed). Similar to our results, there is some evidence that individuals who are worried about becoming infected tend to be diligent and compliant with containment measures [15]. This concept was demonstrated by a global survey, conducted in 48 countries, indicating that people who worried about getting sick were also more likely to be compliant with preventative measures, and tended to trust their government’s recommendations and policies the most [15]. In addition, some researchers also suggested leveraging negative emotional appeals to reach audiences that may be emotionally disengaged or apathetic about vaccination, to increase their knowledge of COVID-19 risk and severity, and finally increase vaccine uptake [14,22]. Fear appeals might be a persuasive message that attempts to get people to adopt recommendations by highlighting the potential danger and harm that would occur if they don’t heed the message’s advice [23]. Our results were consistent with the above theories. The association between emotions and health behaviors is complex. There exists heterogeneity in the emotional responses to messages of public health interventions, and a message intending to activate one kind of emotion may not have the desired effect [14,32]. Accordingly, a message-behavior-audience framework for communication effectiveness was put forward, taking into account the content of the message, the nature of the behavior recommended by the communication, and the characteristics of the audience receiving the message [22]. Even though our analysis was cross-sectional and could not evaluate change over time, our findings suggest that promoting negative emotions about COVID-19 to persuade people to get vaccinated could potentially increase their willingness to do so. However, such an approach raises ethical considerations. Deontologists would argue that regardless of the societal gains it is wrong to cause anxiety and stress in a population. While teleologists would posit that the ends can justify the means if those ends are socially beneficial. The resolution of this ethical debate is certainly beyond the scope of this manuscript but is important to consider for those thinking about future COVID-19 vaccination campaigns.

People nowadays have increased their knowledge about COVID-19 and the related preventive measures in different age groups of people in European countries, including the elderly and the young [5,33]. A higher knowledge level of the disease (including transmission, treatment, and preventive methods) was also associated with higher COVID-19 vaccine acceptance [13,34]. Accompany the spread of knowledge, increased mental health issues, such as stress, anxiety, depressive symptoms, insomnia, denial, anger, and fear, have been reported globally during the COVID-19 pandemic [35]. A population-based study conducted in Sweden between May and June 2020, at the height of the COVID-19 pandemic, reported around 40% of respondents had significant problems in one or more areas of their mental health, including depression, anxiety, or insomnia [17]. Another study showed that children and adolescents were also worried about the pandemic for themselves and their elderly relatives [36]. Interestingly, a Swedish study that used crowdsourced, online data and qualitative analysis found that the most salient population concerns during COVID-19 were centered on a range of social problems, including fear of general societal collapse and political disorder [37]. Our study further found that those having experienced economic stress-due to concerns of not being able to afford enough food-were less likely to accept the vaccine. The finding is consistent with previous studies that demonstrated that higher income is associated with higher vaccine acceptance [11]. During the pandemic, in addition to physical and mental health issues, people have experienced financial challenges. Due to the first year of the pandemic, the Swedish economy contracted by approximately 2%, and the unemployment rate reached 8% [38]. Balancing policies limiting economic disruptions while controlling the spread of the virus has been challenging for all nations due to the uncertainty of the situations and the multiple factors driving the decision-making process. When decisions are made based on limited information, it is sometimes difficult to communicate to the public in a transparent manner, which may influence public opinions about the government’s response. Interestingly, our study found that respondents with negative opinions about the government’s response to the pandemic were less likely to accept the vaccine, consistently with other studies [39].

In terms of practical recommendations derived from this study, our results suggest that reducing the economic stress experienced by individuals during a crisis may have a positive impact on compliance with COVID-19 immunization behaviors. Our results also suggest that understanding what aspects of the government’s response are perceived as ineffective by the public, and eventually improving communication about the decision-making process around such aspects, may also have a positive impact on immunization compliance. Persuasion techniques leading people to feel more worried about the situation may work as well, but need to be addressed in ethical terms. Care should be taken not to allay negative concerns about the severity of the pandemic. For future communication campaigns, communication strategies, in which the role of emotions, economic stress, and opinions about the government response are taken into consideration, may be effective in reaching those that are still hesitant about the vaccine. Findings from our study might be helpful to policymakers in weighing the implications of different response measures and communication strategies supporting such measures.

This study presents some limitations. Firstly, because of the cross-sectional design, due to the lack of temporal ordering of our observed associations between negative emotions and vaccine acceptance, we cannot claim that they are causal in nature. Second, our sample was not representative of the Swedish population, and as such, our results cannot be generalized beyond the sample characteristics. Participants were smartphone users who may have different exposure to information compared to other segments of the population. Finally, in absence of validated scales on negative emotions related to the COVID-19 pandemic, we created our own based on existing literature and included some reported negative emotions and the corresponding objects of concern [1,2,3,17]. However, the scale may not fully capture the multiple aspects of this complex construct. Furthermore, our scale is not intended to diagnose anxiety or depression, and while our construct may be conceptually related to anxiety and depression, we make no claims to its predictive or concurrent validity to assess it clinically.

## 5. Conclusions

People who reported strong negative emotions (feeling anxious, depressed, upset, and stressed) toward COVID-19 were more willing to get vaccinated. On the contrary, individuals having experienced economic stress and those with negative opinions about the government’s response to the pandemic were less willing to get the vaccine. Our results suggest that policies that reduce the economic stress experienced by individuals may have an impact on vaccine acceptance. Our results also indicate that future research should focus on understanding what aspects of the government’s response are perceived as ineffective by the public and eventually improving communication on the decision-making process leading to specific policies, which may also have a positive impact on immunization practices.

## Figures and Tables

**Table 1:** Baseline characteristics and their relationships with vaccine acceptance.

Characteristics	Overall (N = 965) N (%)	Acceptant (N = 494) N (%)	Hesitant (N = 471) N (%)	*p*
**Sex**				
Male	477 (49.4)	242 (50.7)	235 (49.3)	0.778
Female	488 (50.6)	252 (51.6)	236 (48.4)	
**Age**				
18–34	383 (39.7)	163 (42.6)	220 (57.4)	<0.001 **
35–44	195 (20.2)	83 (42.6)	112 (57.4)	
45–54	192 (19.9)	111 (57.8)	81 (42.2)	
Over 54	195 (20.2)	137 (70.3)	58 (29.7)	
**Employment status**				
Unemployed	300 (31.1)	150 (50.0)	150 (50.0)	0.619
Employed	665 (68.9)	344 (51.7)	321 (48.3)	
**Education**				
Less than high school	101 (10.5)	44 (43.6)	57 (56.4)	0.167
High school	463 (48.0)	234 (50.5)	229 (49.5)	
College and above	401 (41.6)	216 (53.9)	185 (46.1)	
**Economic stress**				
No	620 (64.2)	356 (57.4)	264 (42.6)	<0.001 **
Yes	345 (35.8)	138 (40.0)	207 (60.0)	
**Comorbidities**				
No	602 (62.4)	281 (46.7)	321 (53.3)	0.001 **
One comorbidity	245 (25.4)	145 (59.2)	100 (40.8)	
Two or more comorbidities	118 (12.2)	68 (57.6)	50 (42.4)	
**COVID-19 diagnosis**				
No	772 (80.0)	415 (53.8)	357 (46.2)	0.001 **
Yes	193 (20.0)	79 (40.9)	114 (59.1)	
**Vaccination refusal in the past**				
No	691 (71.6)	371 (53.7)	320 (46.3)	0.014 *
Yes	274 (28.4)	123 (44.9)	151 (55.1)	
**Opinions about the government’s COVID-19 response**				
Not right	699 (72.4)	314 (44.9)	385 (55.1)	<0.001 **
Just right	266 (27.6)	180 (67.7)	86 (32.3)	
**Negative emotion degree**				0.009 **
Low	348(36.1)	158(45.4)	190(54.6)	
Medium	329(34.1)	170(51.7)	159(48.3)	
High	288(29.8)	166(57.6)	122(42.4)	

**: *p* < 0.01, *: *p* < 0.05.

**Table 2:** Logistic Regression Models for COVID-19 Vaccine Acceptance.

Variables	Model 1		Model 2		Model 3	
	OR(SE)	95% CI	OR(SE)	95% CI	OR(SE)	95% CI
**Sex**						
Male	Ref	-	Ref	-	Ref	-
Female	1.13 (0.16)	0.86–1.48	1.14 (0.16)	0.86–1.50	1.09 (0.16)	0.82–1.45
**Age**						
18–34	Ref	-	Ref	-	Ref	-
35–44	1.11 (0.20)	0.78–1.58	1.02 (0.19)	0.71–1.46	1.00 (0.19)	0.69–1.47
45–54	2.14 (0.40) **	1.49–3.08	1.98 (0.37) **	1.37–2.86	1.74 (0.34) **	1.19–2.55
Over 54	3.79 (0.74) **	2.58–5.55	3.41 (0.69) **	2.30–5.06	2.82 (0.59) **	1.87–4.25
**Employment status**						
Unemployed	-	-	Ref	-	Ref	-
Employed	-	-	0.99 (0.16)	0.73–1.35	1.07 (0.18)	0.78–1.47
**Education**						
Less than high school	-	-	Ref	-	Ref	-
High school	-	-	1.26 (0.30)	0.79–2.00	1.31 (0.32)	0.82–2.11
College and above	-	-	1.31 (0.32)	0.81–2.11	1.33 (0.33)	0.81–2.18
**Economic stress**						
No	-	-	Ref	-	Ref	-
Yes	-	-	0.46 (0.07) **	0.34–0.62	0.43 (0.07) **	0.31–0.59
**Comorbidities**						
No	-	-	-	-	Ref	-
One comorbidity	-	-	-	-	1.68 (0.29) **	1.20–2.36
Two or more comorbidities	-	-	-	-	1.61 (0.38) *	1.02–2.54
**COVID-19 diagnosis**						
No	-	-	-	-	Ref	-
Yes	-	-	-	-	0.59 (0.11) **	0.41–0.84
**Vaccination refusal in the past**						
No	-	-	-	-	Ref	-
Yes	-	-	-	-	0.70 (0.11) *	0.52–0.96
**Opinions about government’s COVID-19 response**						
Not right	-	-	-	-	Ref	-
Just right	-	-	-	-	2.48 (0.40) **	1.80–3.40
**Negative emotion degree**						
Low	Ref	-	Ref	-	Ref	-
Medium	1.45 (0.23) *	1.05–1.99	1.65 (0.27) **	1.19–2.28	1.75 (0.30) **	1.25–2.45
High	2.02 (0.35) **	1.44–2.83	2.51 (0.46) **	1.75–3.60	2.71 (0.52) **	1.86–3.94

OR: odds ratio, SE: standard error, CI: confidence interval, Ref: reference. Model 1: negative emotion and demographic factors (sex and age). Model 2: Model 1+ socioeconomic status factors (education, employment status, and economic stress). Model 3: Model 2+ other risk factors (comorbidities, COVID-19 diagnosis, past vaccination refusal, opinions about the government’s COVID-19 response). **: *p* < 0.01, *: *p* < 0.05.

**Table 3:** Predictive models assessment.

Models		AUC		AIC
		Statistic (95% CI)	*p*	
Model 1	−	0.624 (0.589–0.659)	reference	1295.0
	+	0.651 (0.617–0.685)	0.010 *	1282.1
Model 2	−	0.657 (0.623–0.691)	reference	1282.0
	+	0.681 (0.648–0.715)	0.016 *	1259.5
Model 3	−	0.699 (0.666–0.731)	reference	1236.6
	+	0.723 (0.691–0755)	0.003 **	1211.8

Model 1: Adjusting for demographic factors (including sex and age). Model 2: Adjusting for demographic and socioeconomic status factors (including education, employment status, and economic stress). Model 3: Adjusting for demographic and socioeconomic status factors, and other risk factors (including comorbidities, COVID-19 diagnosis, vaccination refusal in the past, and opinions about the government’s COVID-19 response). +: With negative emotion degree in the model. −: Without negative emotion degree in the model. AUC: area under the receiver operating characteristic curve, AIC: Akaike information criterion, CI: confidence interval. **: *p* < 0.01, *: *p* < 0.05.

## Data Availability

The data are available from the corresponding author upon reasonable request.

## Supplementary Materials

The following supporting information can be downloaded at: https://www.mdpi.com/article/10.3390/vaccines10101695/s1, Table S1. Factor Analysis Results. Table S2. ORs between negative emotion degrees and COVID-19 vaccine acceptance across age groups.
