# Supplementary material for: Exploring the Association between Negative Emotions and COVID-19 Vaccine Acceptance: A Cross-Sectional Analysis of Unvaccinated Adults in Sweden
Source: Vaccines (Basel). 2022 Oct 11;10(10):1695. doi: 10.3390/vaccines10101695 (PMC9608178; doi:10.3390/vaccines10101695)
Supplement: Supplementary file 1 [file vaccines-10-01695-s001.zip › vaccines-1887693-supplementary.pdf]

Supplementary Table S1. Factor Analysis Results.

Item	Factor Loading
Negative Emotions - Factor 1	
1. I feel anxious when I see the number of COVID-19 cases climbing	0.74
2. I feel depressed about the uncertainty of how this pandemic will evolve	0.81
3. I get upset when I hear contradictory information about COVID-19	0.73
4. I feel stressed when I am unable to plan my life due to COVID-19	0.74

Supplementary Table S2. ORs between negative emotion degrees and COVID-19 vaccine acceptance across age groups.

Age group	Negative emotion degree	OR(SE) ◆	95% CI
18-34 (N=383)	Low	Ref	-
	Medium	1.28(0.38)	0.72–2.28
	High	2.29(0.68)	1.28–4.09
35-44 (N=195)	Low	Ref	-
	Medium	2.40(0.91)	1.14–5.03
	High	4.95(2.25)	2.03–12.07
45-54 (N=192)	Low	Ref	-
	Medium	1.35(0.53)	0.62–2.93
	High	2.02(0.93)	0.83–4.96
Over 54 (N=195)	Low	Ref	-
	Medium	2.62(1.09)	1.16–5.94
	High	3.62(1.86)	1.32–9.91

OR: odds ratio, SE: standard error, CI: confidence interval. ◆: Adjusting for sex, employment status, educational level, economic stress, comorbidities, previous COVID-19 diagnosis, past refusal of other types of vaccine, and opinion about the government’s response to the pandemic.
